# Physiological Characterization of Young ‘Hass’ Avocado Plant Leaves Following Exposure to High Temperatures and Low Light Intensity

**DOI:** 10.3390/plants10081562

**Published:** 2021-07-29

**Authors:** Or Shapira, Simon Chernoivanov, Itamar Neuberger, Shay Levy, Lior Rubinovich

**Affiliations:** 1 Fruit Tree Sciences, Agricultural Research Organization (ARO), The Volcani Center, HaMaccabim Road 68, P.O. Box 15159, Rishon LeZion 7528809, Israel; shapirao@volcani.agri.gov.il; 2 Fruit Tree Sciences, Agricultural Research Organization (ARO), Newe Ya’ar Research Center, P.O. Box 1021, Ramat Yishay 30095, Israel; 3 Northern R&D, MIGAL–Galilee Research Institute, P.O. Box 831, Kiryat Shmona 11016, Israel; simonc@migal.org.il (S.C.); itamar.neu@gmail.com (I.N.); shayl@migal.org.il (S.L.)

**Keywords:** heat stress, carbon assimilation, *Persea americana*, stomatal conductance, subtropic, leaf damage

## Abstract

The worldwide demand for avocados has resulted in the planting of millions of young plants each year. However, global warming, resulting in high temperatures, sensed as heat stress, may severely damage these new plantings. The objective of this study was to assess the risks of heat stress on young avocado plants. We aimed to characterize different physiological parameters of young ‘Hass’ plant leaves following exposure to high temperatures under low light (LL) intensity and to pinpoint the temperature threshold for significant heat stress damage in these plants. To this end, young potted plants were subjected to different temperature gradients in a controlled-climate chamber. Minor and severe leaf damage was apparent in plants subjected to the 51 °C and 53 °C treatments, respectively. Minor and vast reductions in optimal quantum yield efficiency of photosystem II (Fv/Fm) values were observed in plants subjected to 51 °C and 53 °C, respectively. Heat stress treatments significantly reduced CO_2_ assimilation in plants subjected to 49 °C and higher temperatures. Stomatal conductance to water vapour and substomatal internal CO_2_ concentration were less sensitive to the heat treatments. These results imply that the heat damage threshold for young avocado plants under LL conditions is between 49 °C and 51 °C, whereas at 53 °C, severe and irreversible leaf damage occurs.

## 1. Introduction

Over the last decade, changing climate patterns have been associated with global warming [1]. Plants may be severely damaged by very high temperatures, sensed as heat stress [2]. Heat stress significantly affects plant developmental processes such as seed germination, vegetative growth and reproductive production [3]. Moreover, heat stress may impair crucial physiological processes in the plant, namely photosynthesis and respiration rates, stomatal conductance and leaf water potential homeostasis [4]. At high temperatures, net carbon assimilation may decline due to various processes such as increased photorespiration, increased mitochondrial respiration, inactivation of Rubisco and decreased activity of photosystem II [5,6]. Stomatal conductance may be elevated or reduced, depending on the plant species and the heat stress conditions [7]. Heat stress may also reduce the optimal quantum yield of photosystem II (Fv/Fm) ratio, which leads to significant reductions in photosynthesis [3,8]. Other direct effects of heat stress, such as increased fluidity of the thylakoid membranes, increased reactive oxygen species (ROS) production, inactivation of enzymes, loss of membrane integrity, inhibition of protein regulations and protein degradation may also lead to severe injuries to different plant tissues [9]. Eventually, visible foliar damage may occur, depending on the duration and the timing of the exposure to the high temperatures [10]. As heat stress may severely limit the productivity of various commercially important crops, global warming has potential calamitous impacts on global food security [3,11]. 

In the last few years, the avocado (*Persea americana* Mill.) has become a commercially important subtropical fruit crop in many countries worldwide [12]. The avocado cv. ‘Hass’, a black-skinned hybrid of the Guatemalan and Mexican landraces, accounts for over 85% of global avocado commerce [13]. As the demand for avocado fruit rises worldwide, millions of young avocado plants are being planted each year in countries with tropical and subtropical climates [14]. However, as global warming is resulting in more frequent extreme climatic events, these new plantings may be severely damaged by very high temperatures in the young orchards [15]. For example, in the spring of 2020, an extreme heat wave occurred in Israel. In the Jordan Valley, one of the avocado cultivation regions in Israel, maximum daily temperatures exceeded 45 °C in the shade (https://ims.data.gov.il/, accessed on 14 July 2021). 

Numerous studies have addressed the effects of heat stress on fruit trees, including citrus, litchi, mango, and others [4]. Although several studies have assessed the effect of heat stress during flowering and early fruit set on mature avocado trees [16,17], very little is known of its effects on young avocado plants. Therefore, in the light of climate change and the gradual increase in global temperatures which may limit avocado cultivation and orchard expansion, the main objective of this study was to assess the risk from heat stress on young avocado plants. Specifically, we aimed to characterize different physiological parameters of young ‘Hass’ avocado plants following exposure to high temperatures and to pinpoint the temperature threshold for significant heat stress damage in these plants. As combination of two different abiotic stresses, such a heat stress and high light intensity, will result in more tissue damage than that obtained with each stress separately [9,18]. In this study we examined the effect of heat stress with low light (LL) intensity. That way, we could isolate and focus on the effect of heat stress on the plants, rather than the combined effect of two different abiotic stresses. The main hypothesis of this study is that young avocado plants will be gradually damaged with the increase of laboratory high temperature treatments, up to a certain yet-unknown threshold, where plants will be severely and irreversibly damaged. 

## 2. Results

**Figure 1:**
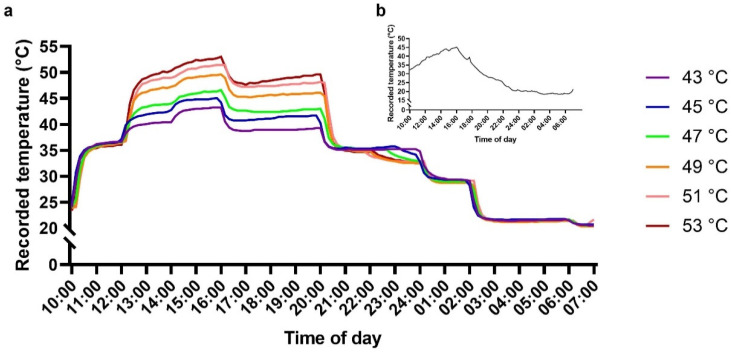
Artificial and natural temperature gradients. (**a**) Avocado plants were subjected to the different stepped temperature gradients recorded in the controlled-climate chamber. (**b**) Recorded temperatures of an actual extreme heat event that occurred in July 2020 in an avocado orchard located in northern Israel.

To characterize the effect of heat stress on physiological properties of young ‘Hass’ avocado plants, potted plants were subjected to 43 °C, 45 °C, 47 °C, 49 °C, 51 °C and 53 °C treatments. Although natural field daily temperatures are characterized by a sloped gradient (Figure 1a), the climate chamber could create only stepped temperature gradients (Figure 1b). Still, both natural and artificial temperature gradients were very similar. 

### 2.1. Effect of Heat Stress on Leaf Damage 

**Figure 2:**
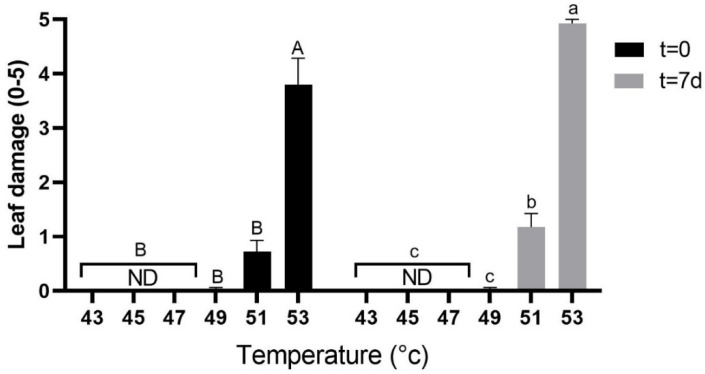
Leaf damage assessment. Leaf damage was assessed and scored on a scale of 0–5, with 0 representing no apparent damage and 5, maximum damage. ND–not detected. Measurements were taken right after climate chamber incubation and before exposure to sunlight (t = 0), and after seven days in the net house (t = 7 d). Each treatment was replicated four times with four different plants for each replicate. Values are means ± SE of at least four different leaves on each of the 16 plants (*n* = 16). Columns marked with different letters differ significantly (Tukey-HSD, *p* < 0.05); uppercase letters for t = 0 and lowercase letters for t = 7 d.

At t = 0, leaf damage was not apparent following the 43 °C, 45 °C and 47 °C treatments (Figure 2 and Figure S1). Minor damage started to show on leaves from plants subjected to the 49 °C and 51 °C treatments. Plants subjected to the 53 °C treatment showed significantly greater damage. Similar trends and leaf damage assessments were seen at t = 7 d. 

### 2.2. Effect of Heat Stress on Optimal Quantum Yield of Photosystem II (Fv/Fm)

**Figure 3:**
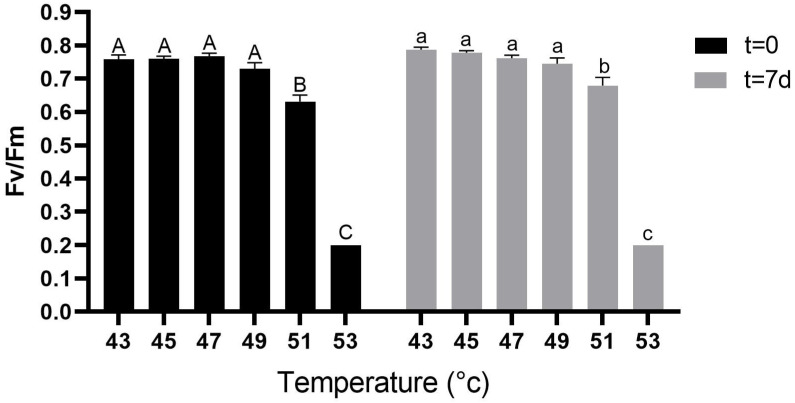
Quantum yield of photosystem II (Fv/Fm) calculated from chlorophyll *a* fluorescence recorded after dark adaptation. Measurements were taken right after climate chamber incubation and before exposure to sunlight (t = 0), and pre-dawn after seven days in the net house (t = 7 d). Each treatment was replicated four times with four different plants for each replicate. Values are means ± SE of at least four different leaves on each of the 16 plants (*n* = 16). Columns marked with different letters differ significantly (Tukey-HSD, *p* < 0.05); uppercase letters for t = 0 and lowercase letters for t = 7 d.

Fv/Fm rates before the heat treatments, at t = −1 d, ranged between 0.77 and 0.81 (Figure S2) and were similar between the plants which were subsequently used for all the different treatments. Fv/Fm values at t = 0 were high in plants subjected to the 43 °C, 45 °C, 47 °C and 49 °C treatments and ranged between 0.73 and 0.77 (Figure 3), significantly higher than for plants subjected to the 51 °C treatment (0.63); the latter value was significantly greater than that of plants subjected to the 53 °C treatment (0.2). Similar trends of Fv/Fm were still evident at t = 7 d (Figure 3).

### 2.3. Effect of Heat Stress on Gas-Exchange Parameters

**Figure 4:**
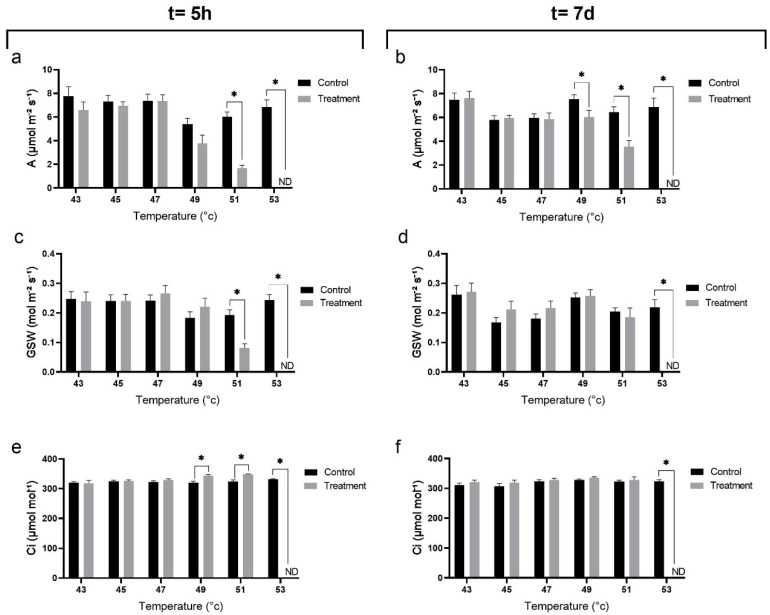
Gas-exchange parameters were measured after exposure to sunlight following temperature treatments: A–CO_2_ assimilation, GSW–stomatal conductance to water vapour, and Ci–substomatal internal CO_2_ concentration. Measurements were taken after incubation in the climate chamber (treatment), following five hours of exposure to sunlight in the net house (**a**,**c**,**e**; t = 5 h), and at midday after seven days in the net house (**b**,**d**,**f**; t = 7 d). Plants that were not exposed to the different heat treatments were used as control (control). Each treatment was replicated four times with four different plants for each replicate. Values are means ± SE of at least four different leaves on each of the 16 plants (*n* = 16). Columns marked with an asterisk differ significantly (Student’s *t*-test, * *p* < 0.05).

In general, plants that were not exposed to the different heat treatments (control) assimilated ~6–8 μmol CO_2_ m^−2^ s^−1^ (Figure 4a,b) with variations caused mainly by changing weather on the measurement day and daily effects. At t = 5 h, heat treatments (treatment) significantly affected CO_2_ assimilation only in plants subjected to the 51 °C and higher treatments. Following the 53 °C treatment, assimilation was not detected due to severe leaf damage (Figure 4a). At t = 7 d, assimilation was similar to that of the control in the 43 °C, 45 °C and 47 °C treatments (Figure 4b). In the 49 °C and the 51 °C treatments, assimilation was slightly higher than at t = 5 h, but was significantly lower than in the control. In the 53 °C treatment, assimilation was still not detected. Stomatal conductance to water vapour (GSW) at t = 5 h was less sensitive to the heat treatments, showing a reduction compared to the control only after exposure to the 51 °C and 53 °C (Figure 4c). At t = 7 d, only plants that were subjected to the 53 °C treatment had no detectable GSW, while full recovery was detected in plants subjected to the 51 °C treatment (Figure 4d). 

At both time points, substomatal internal CO_2_ concentration (Ci) in the control plants was slightly above 300 µmol mol^−1^. At t = 5 h, Ci increased significantly in plants subjected to the 49 °C and 51 °C treatments, as compared to the control plants, whereas in plants subjected to the 53 °C treatment, there was no assimilation and Ci was not detected (Figure 4e). At t = 7 d, there was no difference in Ci between the control and any of the heat treatments, except in plants subjected to the 53 °C treatment, where Ci remained undetectable (Figure 4f).

## 3. Discussion

In this study, we characterized physiological parameters of young ‘Hass’ avocado plants following exposure to different high-temperature treatments. The climate chamber temperature gradients were designed to simulate actual field temperature gradients. 

First signs of heat stress damage to the leaves were already observed with the 49 °C treatment, as CO_2_-assimilation rates were lower than in the control. However, no apparent leaf damage or decrease in Fv/Fm values were observed at this temperature, suggesting only a mild effect of the treatment. There was a significant decrease in Fv/Fm values and in gas-exchange parameters followed by clearly observable leaf damage at 51 °C, implying that the heat damage threshold for young avocado plants is between 49 °C and 51 °C. The reduction in Fv/Fm values and the CO_2_-assimilation rate persisted one week later, at t = 7 d, suggesting that this damage is irreversible, at least during the time period of the experiment.

When plants were subjected to a maximum temperature of 53 °C, significant and irreversible heat stress damage resulted in severe leaf damage, very low Fv/Fm values and zero assimilation rate. Although extreme climatic events with air temperature reaching 53 °C are currently rare in avocado-growing locations, a significant decrease in plant physiological parameters was already observed in the lower temperature heat stress treatments. It should also be noted that the combination of different abiotic stresses may result in more tissue damage than that obtained with each stress separately [18,19,20]. For example, heat stress and high light intensity are both abiotic conditions that can separately impact the photosynthetic machinery performance. The combination of these two stresses has unique physiological and molecular characteristics and would have a severe effect on photosystem II integrity and activity, higher than that of each individual stress [9]. In fact, the major difference between the heat stress imposed in the controlled-climate chamber and actual heat stress in the field is the relatively LL intensity in the former (100 μmol m^−2^ s^−1^). Therefore, under field conditions, where high temperatures and high light intensity occur at the same time, substantial damage is expected to occur at even lower temperatures than those that were found in this study. 

Fv/Fm, the optimal quantum yield of photosystem II, is a chlorophyll *a* fluorescence parameter that allows for a rapid assessment of the entire light-harvesting system, excess-energy dissipation and assimilation processes [21]. When regulated energy dissipation is not sufficient under stress conditions, chronic photodamage manifests as a significant reduction in Fv/Fm. The fact that significant damage to photosystem II occurred only from 51 °C, but not at temperatures lower than 49 °C suggests that the latter temperature is the lower threshold for heat stress damage. It is important to note that the damage occurred during the treatments and not as a result of exposure to natural light immediately after the treatment, as plants were subjected to a light intensity of only ~100 μmol m^−2^ s^−1^, as noted above. 

The CO_2_-assimilation rate in the young avocado plants ranged between 5 and 8 μmol m^−2^ s^−1^, similar to previously published results for young potted ‘Hass’ plants [22,23]. CO_2_ assimilation was only significantly reduced at 49 °C and above, suggesting this as the temperature threshold for heat stress damage in young avocado plants. The significant increase in Ci supports these results, as this value represents a reduction in the ability to use available CO_2_ in the substomatal cavity. The differences in damage thresholds for Fv/Fm vs. CO_2_ assimilation might be due to the resilience of the light-harvesting system compared to the CO_2_-assimilation machinery [24,25]. GSW may have been less sensitive to the heat stress than CO_2_ assimilation because of the passive nature of the hydraulic system compared to the enzymatic nature of the photosynthetic machinery [26]. 

In a wider scope, as the frequency of extreme weather events which combine high temperatures with high-light intensity conditions increase dramatically [9], significant effort should be invested into finding solutions to mitigate heat stress in avocado plants. For example, in the short term, the use of shading nets to reduce heat and irradiance [27] and cooling of the tree canopy using water evaporation might be examined [28]. In the long term, heat stress characterization may be implemented in ongoing commercial avocado breeding programs to develop elite heat-resilient cultivars [5].

In conclusion, this study characterized the effects of heat stress on young potted avocado plants. Our hypothesis was that young avocado plants will be gradually damaged with the increase of the controlled high temperature treatments, up to a certain threshold, where they will be severely and irreversibly damaged. Indeed, the plants were gradually damaged from 49 °C, until severely and irreversibly damaged at 53 °C. Thus, our results suggest that the heat stress threshold for young ‘Hass’ avocado plants is between 49–51 °C under LL intensity. We also assume that this threshold will be lower at field conditions, where irradiance levels are usually much higher. In order to determine this threshold, further research is needed to characterize short and long term effects of combined heat stress and high-light stress under controlled and field conditions. 

## 4. Materials and Methods

### 4.1. Plant Material

One-year-old ‘Hass’ avocado plants (growing in 7-L pots) grafted on ‘Degania 117’ rootstock were chosen for this study. Plants were grown in an outdoor net house with an average photosynthetic photon flux density (PPFD) of 400–450 μmol m^−2^ s^−1^. Plants were drip irrigated every morning until significant drainage.

### 4.2. Heat-Stress Treatments and Temperature Measurements 

Plants were subjected to artificial heat stress by exposing them to six different high temperature gradients for ~24 h in a Nuve TK600 climate chamber (Ankara, Turkey). Air temperature data were recorded continuously at 10 min intervals and collected by a miniature, waterproof, single-channel Hobo temperature data logger (UA-001-64; Onset Corp., Bourne, MA, USA). Actual temperatures of the different heat stress gradients peaked at ~43 °C, 45 °C, 47 °C, 49 °C, 51 °C and 53 °C (Figure 1a). Gradients were designed to mimic actual extreme heat events, such as that which occurred during July 2020 in an avocado orchard located in northern Israel (Figure 1b). The soil and root system were insulated from the heat by covering them with heat isolating Styrofoam. Constant fluorescent PPFD of ~100 μmol m^−2^ s^−1^ (LL) was applied inside the controlled-climate chamber for the first 12 h of each treatment to simulate daytime, and was turned off until the end of the treatment to simulate night. Following the heat stress treatments, plants were transferred back to the net house and exposed to ambient conditions. 

### 4.3. Leaf Measurements 

For each plant, leaf measurements were taken and averaged from at least four mature leaves. All measured leaves were positioned four to five leaves from the branch tip. Each temperature treatment and the control treatment were independently replicated four times (with four different plants for each replicate) and data are presented as a grand mean from all 16 different plants which were exposed to the same temperature regime (*n* = 16). 

#### 4.3.1. Leaf Damage Assessment 

Leaf damage was assessed with a blind test in which two surveyors independently scored each leaf on a scale of 0–5, with 0 representing no apparent leaf damage and 5 representing maximum leaf damage [29]. 

#### 4.3.2. Chlorophyll a Fluorescence Analysis

Chlorophyll *a* fluorescence-derived Fv/Fm (optimal quantum yield efficiency of photosystem II) was measured with a FluorPen FP100 portable fluorometer (Photon Systems Instruments, Drasov, Czech Republic) following dark adaptation [21,30,31]. Measurements were taken in the morning before the heat treatment (t = −1 d), right after climate chamber incubation and before exposure to sunlight (t = 0), and pre-dawn after seven days in the net house (t = 7 d). 

#### 4.3.3. Gas-Exchange Measurements

Leaf-level CO_2_ assimilation and leaf-level transpiration were measured using a LI-6800 clear-top portable photosynthesis system (9 cm^2^, LI-COR, Lincoln, NE, USA). Flow was set to 1000 μmol s^−1^ and boundary layer conductance to water vapour was ~3 mol m^−2^ s^−1^ by setting the mixing fan to 10,000 rpm. Measurements were performed in the net house at midday, on mature attached leaves. Climatic conditions in the LI-COR leaf chamber were set to ambient with air temperature averaging 33.04 ± 3.34 °C. Only leaves that were facing the sun at the time of the measurement were measured. Care was taken not to change their orientation to the sun while inside the LI-COR leaf chamber. PPFD recorded inside the LI-COR leaf chamber during all measurements averaged 449 ± 107.8 μmol m^−2^ s^−1^. Stomatal conductance to water vapour (GSW) and substomatal internal CO_2_ concentration (Ci) were calculated by the LI-COR machine. To assess post heat stress damage, measurements were taken after incubation in the climate chamber, following 5 h of exposure to sunlight in the net house (t = 5 h), and at t = 7 d. The heat-stressed plants were always compared to control plants (control) from the same batch that remained in the net house for the duration of the treatment and were not exposed to the high temperature treatments.

### 4.4. Statistical Analysis

All leaf damage and Fv/Fm results from the same measurement time points were subjected to one-way analysis of variance (ANOVA) followed by Tukey-HSD test in JMP version 11.0.0 (SAS Institute, Cary, NC, USA). All gas-exchange results from the same measurement time points were subjected to ANOVA with a single heat stress factor. Means for control vs. heat stress pairs were separated by Student’s t-test using the same software.

## Data Availability

Not applicable.

## Supplementary Materials

The following are available online at https://www.mdpi.com/article/10.3390/plants10081562/s1, Figure S1: Leaf damage assessment. Representative pictures of potted ‘Hass’ avocado plants subjected to the different high-temperature treatments. Pictures were taken right after climate chamber incubation and before exposure to sunlight (t = 0) and at midday after seven days in the net house (t = 7 d), Figure S2: Quantum yield of photosystem II (Fv/Fm) calculated from chlorophyll a fluorescence recorded after dark adaptation. Measurements were taken in the morning before the heat treatments at t = −1 d. Temperatures indicated on the X-axis refer to heat treatments conducted the following day. Each treatment was replicated four times with four different plants for each replicate. Values are means ± SE of at least four different leaves on each of the 16 plants (n = 16).
