# Supplementary material for: Physiological Characterization of Young ‘Hass’ Avocado Plant Leaves Following Exposure to High Temperatures and Low Light Intensity
Source: Plants (Basel). 2021 Jul 29;10(8):1562. doi: 10.3390/plants10081562 (PMC8400502; doi:10.3390/plants10081562)
Supplement: Supplementary file 1 [file plants-10-01562-s001.zip › plants-1319859-supplementary.pdf]

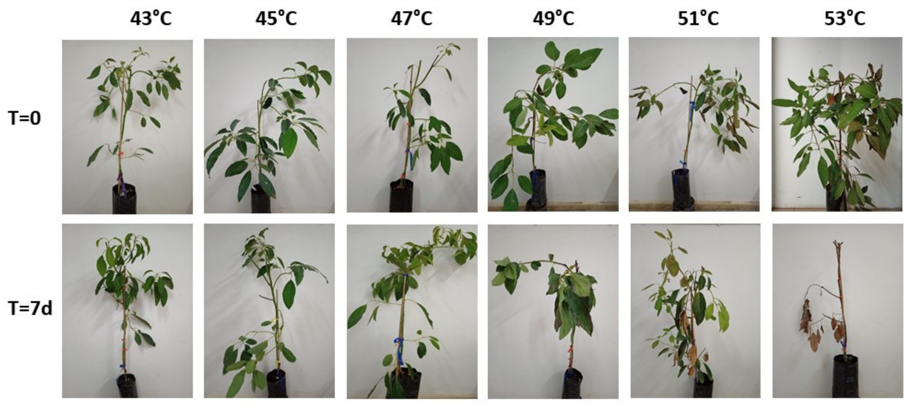

Figure S1. Leaf damage assessment. Representative pictures of potted 'Hass' avocado plants subjected to the different high-temperature treatments. Pictures were taken right after climate chamber incubation and before exposure to sunlight (t = 0) and at midday after seven days in the net house (t = 7d).

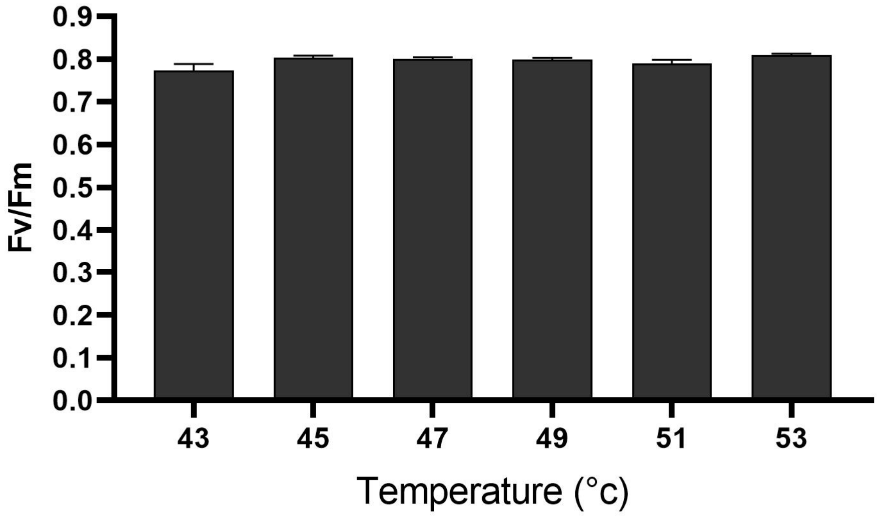

Figure S2. Quantum yield of photosystem II (Fv/Fm) calculated from chlorophyll *a* fluorescence recorded after dark adaptation. Measurements were taken in the morning before the heat treatments at t = -1d. Temperatures indicated on the X-axis refer to heat treatments conducted the following day. Each treatment was replicated four times with four different plants for each replicate. Values are means ± SE of at least four different leaves on each of the 16 plants (n = 16).
